# MiR-192-5p Ameliorates Hepatic Lipid Metabolism in Non-Alcoholic Fatty Liver Disease by Targeting *Yy1*

**DOI:** 10.3390/biom14010034

**Published:** 2023-12-26

**Authors:** Lina Ma, Huichen Song, Chen-Yu Zhang, Dongxia Hou

**Affiliations:** 1 Nanjing Drum Tower Hospital Center of Molecular Diagnostic and Therapy, State Key Laboratory of Pharmaceutical Biotechnology, School of Life Sciences, Nanjing University, Nanjing 210023, China; dg1830021@smail.nju.edu.cn (L.M.); dg1830025@smail.nju.edu.cn (H.S.); 2 Jiangsu Engineering Research Center for MicroRNA Biology and Biotechnology, NJU Advanced Institute of Life Sciences (NAILS), Nanjing 210023, China; 3 Research Unit of Extracellular RNA, Chinese Academy of Medical Sciences, Nanjing 210023, China

**Keywords:** miR-192-5p, NAFLD, *Yy1*, triglyceride metabolism

## Abstract

Non-alcoholic fatty liver disease (NAFLD) is characterized by excessive lipid accumulation in the liver. Clarifying the molecular mechanism of lipid metabolism is crucial for the treatment of NAFLD. We examined miR-192-5p levels in the livers of mice in which NAFLD was induced via a high-fat diet (HFD), as well as in mouse primary hepatocytes and human HepG2 cells treated with free fatty acids (FFAs). MiR-192-5p inhibitor was administered to NAFLD mice and hepatocytes to verify the specific function of miR-192-5p in NAFLD. We validated the target gene of miR-192-5p and further illustrated the effects of this miRNA on the regulation of triglyceride (TG) metabolism. We found that miR-192-5p was significantly increased in the livers of NAFLD mice and FFA-treated hepatocytes. Inhibition of miR-192-5p increased the accumulation of hepatic TGs and aggravated hepatic steatosis in NAFLD mice. In FFA-treated hepatocytes, miR-192-5p inhibitors markedly increased TG content, whereas overexpression of miR-192-5p reduced TG levels. Yin Yang 1 (*Yy1*) was identified as the target gene of miR-192-5p, which regulates TG synthesis via the YY1/fatty-acid synthase (FASN) pathway. Our results demonstrated that miR-192-5p should be considered a protective regulator in NAFLD that can inhibit hepatic TG synthesis by targeting *Yy1*.

## 1. Introduction

Non-alcoholic fatty liver disease (NAFLD) is a prevalent liver disorder worldwide, with an incidence rate of approximately 25% [1]. It is characterized by the accumulation of fat in the liver and is strongly associated with obesity, insulin resistance, and metabolic syndrome [2,3,4]. It represents a spectrum of liver disease that can be simplified into simple steatosis, non-alcoholic steatohepatitis (NASH), and cirrhosis [5]. Simple steatosis (SS), known as fatty liver, is commonly regarded as benign, and it might not cause significant liver damage [3]. However, it can progress to more severe forms of NAFLD if left untreated. NASH is a more advanced form of NAFLD and is characterized by inflammation and liver cell damage in addition to fat accumulation [6]. NASH can lead to liver fibrosis, cirrhosis, and even hepatocellular carcinoma (HCC) [5,6]. In the liver, Triglycerides (TGs) are produced via the esterification of glycerol with fatty acids (FAs), which are chiefly derived from dietary fat, adipose lipolysis, and de novo lipogenesis (DNL) [7,8,9]. In individuals with NAFLD, the DNL rate is more than three times higher than in those without, and the proportion of liver TGs produced by DNL—rather than from adipose tissue lipolysis or dietary FAs—is twice as high [10]. DNL is mediated by three main enzymes: acetyl-CoA carboxylase 1 (ACC1), which catalyzes the synthesis of malonyl-CoA from acetyl-CoA; FA synthase (FASN), which produces palmitic acid and other fatty acid synthesis products from malonyl-CoA; and stearoyl-CoA desaturase 1 (SCD1), which results in the conversion of saturated FAs to monounsaturated FAs [11,12]. DNL is generally believed to play a key role in the progression of NAFLD [13], and therefore DNL inhibition is a potential therapeutic strategy for this disease.

MicroRNAs (miRNAs) are a type of single-stranded non-coding RNAs (ncRNAs) approximately 22 nt long that play a crucial role in the post-transcriptional regulation of gene expression and are involved in multiple physiological processes [14]. Recent investigations have shown that numerous miRNAs are involved in the regulation of hepatic lipid metabolism, including miR-122, miR-33, and miR-125b [15,16,17]. These findings suggest that miRNAs could be potential therapeutic targets in liver metabolic disorders. MiR-192-5p, a highly expressed microRNA in the liver, has been found to regulate hepatocyte differentiation and liver development [18,19]. Furthermore, miR-192-5p plays a significant role in liver pathology, and its dysregulation has been implicated in several liver diseases, including chronic hepatitis B, liver injury, and HCC [20,21,22]. Additionally, serum miR-192-5p levels are significantly elevated in NAFLD patients [23]. However, the precise mechanisms underlying the effects of miR-192-5p on liver lipid metabolism remain unclear.

Yin Yang 1 (YY1) is a member of the glioma-associated oncogene (*GLI*)-Kruppel zinc finger protein family [24,25]. It is a broadly expressed, multifunctional transcription factor (TF) that activates or represses target gene expression at both the transcriptional and post-transcriptional levels [26,27]. Aberrant expression of YY1 is closely associated with various diseases, especially NAFLD. YY1 expression is upregulated in obese mice induced by a high-fat diet (HFD) and in NAFLD patients; the gene promotes hepatic TG synthesis by activating the expression of FASN [28,29]. In this study, we observed a significant upregulation of miR-192-5p in the livers of HFD-induced NAFLD mice compared with those of mice fed a chow diet (CD). However, knockdown of miR-192-5p expression aggravated hepatic steatosis in HFD-induced NAFLD mice. By manipulating miR-192-5p levels in mouse primary hepatocytes and HepG2 cells, we observed that miR-192-5p reduced TG synthesis in hepatocytes. Finally, *Yy1* was verified as a target gene of miR-192-5p via luciferase assay and Western blotting. In summary, our study demonstrated that miR-192-5p acted as a compensatory protector against dyslipidemia by regulating the YY1/FASN pathway, suggesting that this miRNA could be a potential therapeutic target in NAFLD.

## 2. Materials and Methods

### 2.1. Animals

Eight-week-old male C57BL/6J mice were obtained from GemPharmatech Laboratory (Nanjing, China) and maintained on a 12 h light-dark cycle with ad libitum access to food and water. The animal experiment procedures were carried out in accordance with the guidelines set by the Institutional Animal Care and Use Committee (IACUC) and were approved by the Science and Technology Ethics Committee of Nanjing University (Nanjing, China). Mice were subjected to a 16-week experimental period in which they were fed a high-fat diet (HFD) (Cat# D12492, Research Diets, New Brunswick, NJ, USA) to induce the development of non-alcoholic fatty liver disease (NAFLD). During this period, the control group of mice was maintained on a normal chow diet (CD) (Cat# SWS9102, Jiangsu Xietong Pharmaceutical Bioengineering, Nanjing, China).

### 2.2. Lentiviral (LV)-Anti-miR-192-5p Administration

The LV-Anti-miR-192-5p was designed and constructed by GenePharma (Shanghai, China). In brief, Anti-miR-192-5p (GATCCGGCTGTCAATTCATAGGTCAGCGATGGCTGTCAATTCATAGGTCAGTCACGGCTGTCAATTCATAGGTCAGTTTTTGAATT) was cloned into the LV3 (H1/GFP&Puro) vector. The recombinant plasmid was transfected into 293T cells, and the culture medium was collected after 48 h to prepare lentivirus suspension. Then the gradient-diluted lentivirus suspension was infected with 293T cells for 24 h, and the virus titer was calculated according to the fluorescence cell and dilution multiple [30]. In the LV injection of mice study, 100 μL of 1 × 10^9^ TU/mL LV-Anti-miR-192-5p or LV-Anti-NC were intravenously (i.v.) injected into HFD-induced NAFLD mice according to instructions. During lentiviral administration, mice were continually fed a high-fat diet. Two weeks after the injection, the mice were euthanized, and their blood and liver tissues were collected. Liver toxicity was monitored by plasma alanine aminotransferase (ALT) and aspartate aminotransferase (AST) levels as determined by the ALT assay kit (Cat# C009-2-1, Nanjing Jiancheng Bioengineering Institute, Nanjing, China) and the AST assay kit (Cat# C010-2-1, Nanjing Jiancheng Bioengineering Institute, Nanjing, China). Serum triglyceride was determined using a triglyceride assay kit (Cat# A110, Nanjing Jiancheng Bioengineering Institute, Nanjing, China). 

### 2.3. Cell Culture

HepG2 cells and HEK 293T cells were obtained from the Shanghai Institute of Biochemistry and Cell Biology (Shanghai, China). The cells were cultured in DMEM (Cat# 25200072, Gibco, Waltham, MA, USA) containing 10% FBS (Cat# G11-70500, Genial, Denver, CO, USA) and 1% Penicillin/Streptomycin (Cat# 15140148, Gibco, Waltham, MA, USA) at 37 °C and 5% CO_2_. Primary hepatocytes from mice were isolated by a two-step collagenase perfusion according to the standard protocol as previously described [31]. Eight-week-old mice were anesthetized with 1.25% tribromoethanol, and catheters were inserted into the portal vein. The liver was perfused with 10 mL of perfusion solution (Cat# 17701038, Gibco, Waltham, MA, USA) for 3 min, and then digested with 10 mL of digestion solution containing collagenase IV (Cat# 17104019, Gibco, Waltham, MA, USA) for 3 min. After digestion, cells were filtered with 70 μm filters, then centrifuged at 450 rpm for 5 min. DMEM supplemented with 10% FBS and 1% Penicillin/Streptomycin was used for the culture of mouse primary hepatocytes.

### 2.4. miRNA Transfection

Mouse primary hepatocytes or HepG2 cells were transfected with 50 nM miR-192-5p mimics (RiboBio, Guangzhou, China) or 200 nM inhibitors (RiboBio, Guangzhou, China) using Lipofectamine 2000 (Cat# 11668019, Thermo Fisher Scientific, Waltham, MA, USA). Equal scrambled miRNA mimics or inhibitors were used as a negative control. 24 h later, RNA was collected, and 48 h later, protein was collected. 

### 2.5. Lipid Analysis in Cells

The lipid droplet in mouse primary hepatocytes, or HepG2 cells, was visualized by staining with Nile Red (Cat# 72485, Sigma-Aldrich, St. Louis, MO, USA) as previously described [32]. miR-192-5p mimics or inhibitors were transfected into cells, and after 6 h, the cells were cultured with DMEM medium containing 0.4 mM FFAs (oleic acid: palmitic acid = 1:1) or 0.5% BSA for 72 h. The cells were fixed for 30 min using 4% formaldehyde and stained separately with Nile Red and DAPI (Cat# D9542, Sigma-Aldrich, St. Louis, MO, USA) solutions for 10 min each. The fluorescence was measured using a confocal microscope (TCS SP8-MP) (Leica, Heidelberg, Germany). The excitation wavelengths were 405 nm for DAPI and 562 nm for Nile Red. On the other hand, the levels of triglycerides in cells were detected. Cells were homogenized with PBS, and the triglyceride was assayed using a triglyceride assay kit.

### 2.6. Measurement of Triglyceride in the Liver

Triglycerides in the liver were extracted by chloroform and methanol [33]. Each gram of liver tissue was homogenized with a 20-fold volume chloroform: methanol (2:1, V/V) mixture. ddH_2_O was added, and the homogenate was stored on ice for 10 min. Then the homogenate was subjected to centrifugation at a relatively low speed of 2000× *g* for 5 min. This allowed the separation of the homogenate into two distinct phases, and the upper phase was carefully removed and discarded. The organic phases were reextracted as above and then dried using a nitrogen stream. Triglycerides were quantified using a triglyceride assay kit. 

### 2.7. Luciferase Reporter Assay 

The 3′-UTRs of human or mouse *Yy1* containing binding sequences of the miR-192-5p targets or the mutants (GenScript, Nanjing, China) were synthetically inserted into the 3′-UTR region of the pMIR-report luciferase plasmid (Ambion, Waltham, MA, USA). For transfection, reporter constructs (0.1 μg/well), β-gal plasmid (0.1 μg/well, Ambion, Waltham, MA, USA) to normalize experiments for transfection efficiency, and either miR-192-5p mimics (20 pmol/well) or inhibitors (100 pmol/well) were co-transfected into HEK293T cells in 24-well plates. After 24 h, the cells were harvested and lysed using Luciferase Cell Culture Lysis Reagent (Cat#E1531, Promega, Madison, WI, USA). The cell lysates were subjected to three cycles of freezing and thawing in liquid nitrogen, followed by centrifugation at 10,000× *g* for 10 min. Then, 10 μL of the supernatant was utilized to quantify the luciferase activity using a luciferase assay kit (Cat# E1501, Promega, Madison, WI, USA).

### 2.8. Western Blotting 

Proteins were extracted from cells or tissues using lysis buffers (Cat#P0013B, Beyotime, Shanghai, China) containing protease and phosphatase inhibitors (Cat#P1048, Beyotime, Shanghai, China). About 20 μg of denatured proteins were loaded onto a SDS-PAGE gel and separated based on their size. The proteins were transferred from the gel onto a PVDF membrane. The transferred membrane was blocked with 5% non-fat milk to prevent non-specific binding of antibodies. Then primary antibodies were diluted in a blocking buffer and incubated with the blocked membrane. After a washing step in TBST, the secondary antibody was added. The membrane was then washed with TBST and detected using ECL (Cat# E412-02, Vazyme, Nanjing, China). The primary antibodies were used: anti-β-ACTIN (1:2000, Cat# 8457S, CST, Danvers, MA, USA), anti-FASN (1:1000, Cat# 3180S, CST, Danvers, MA, USA), anti-YY1 (1:1000, Cat# SC-7341, SANTA, Dallas, TX, USA), anti-ACC1 (1:1000, Cat# 4190S, CST, Danvers, MA, USA), and anti-SREBP1 (1:1000, Cat# SC-13551, SANTA, Dallas, TX, USA). The protein bands were analyzed using Image J v1.8.0 software (National institute of health, Bethesda, MD, USA).

### 2.9. RNA Isolation and Real-Time Quantitative PCR

Total RNA was extracted from livers and cultured cells using RNAiso Plus (Cat# 9109, Takara, Tokyo, Japan). cDNA was synthesized from total RNA using HiScript III RT SuperMix (Cat# R323, Vazyme, Nanjing, China) and was subjected to quantitative real-time PCR (qPCR) amplification using SYBR Green (Cat# 31000, Biotium, Hayward, CA, USA). β-actin was used to normalize mRNA levels. For miR-192-5p analysis, RT-PCR was performed following a miRNA First Strand cDNA Synthesis Kit (Cat# MR101, Vazyme, Nanjing, China). qPCR was performed with the TaqMan miR-192-5p probe (Cat# 000491, Applied Biosystems, Waltham, MA, USA). U6 snRNA was used to normalize the miR-192-5p level (Cat# 001973, Applied Biosystems, Waltham, MA, USA). Primer sequences are shown in Supplementary Table S1.

### 2.10. Histology

Liver tissues were collected and fixed in 4% formaldehyde overnight at 4 °C. Subsequently, the tissues underwent embedding in paraffin and were then sliced into sections of 4 μm thickness for H&E staining. For Oil Red O staining, frozen liver tissues were cut into sections with a thickness of 10 μm. Following this, the sections were rinsed with 60% isopropanol for a duration of 2 min and subsequently stained with an Oil Red O solution for 5 min. After staining, the sections were again rinsed with 60% isopropanol for a duration of 30 s. The staining images were performed with an Olympus VS120 microscope (Olympus, Tokyo, Japan).

### 2.11. Statistics 

The data were reported as the mean ± standard error of the mean (SEM). To compare statistical differences between two groups, the Student’s *t*-test was employed. For comparing differences among multiple groups, a one-way analysis of variance (ANOVA) was performed. *p* < 0.05 was considered statistically significant, denoted as * *p* < 0.05, ** *p* < 0.01, and *** *p* < 0.001. All statistical analyses were conducted using GraphPad Prism 8 software (GraphPad, La Jolla, CA, USA).

## 3. Results

### 3.1. MiR-192-5p Was Dramatically Increased in the Livers of NAFLD Mice

**Figure 1:**
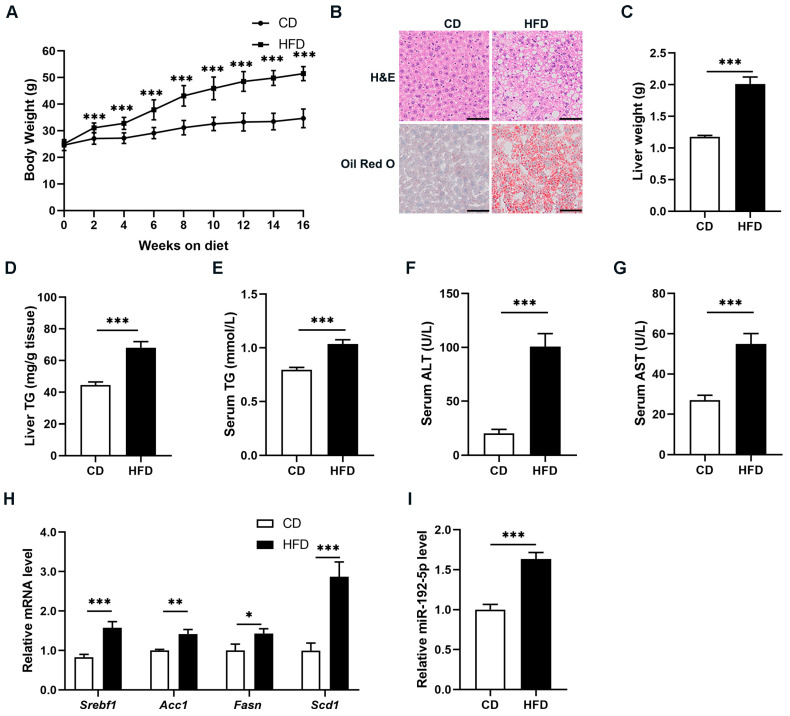
MiR-192-5p was dramatically increased in the livers of NAFLD mice. (**A**) Body weight of high-fat diet (HFD)-induced NAFLD mice and chow diet (CD)-fed control mice (*n* = 10). (**B**) H&E staining (top) and Oil Red O staining (bottom) of liver tissues (Scale bar: 100 μm). (**C**) Weight of livers. (**D**) Hepatic triglyceride (TG) levels. (**E**) Serum TG levels. (**F**) Serum alanine aminotransferase (ALT) levels. (**G**) Serum aspartate aminotransferase (AST) levels. (**H**) Relative expression levels of de novo lipogenesis (DNL)-related genes in livers were measured by qRT-PCR. (**I**) Relative expression levels of miR-192-5p in liver tissues. The data are presented as the mean ± SEM. * *p* < 0.05, ** *p* < 0.01, *** *p* < 0.001.

To investigate the biological effects of miR-192-5p in NAFLD, we first constructed a mouse model of NAFLD via HFD feeding for 16 weeks. As shown in Figure 1A, the body weight of NAFLD mice was significantly higher than that of control mice fed a chow diet (CD). H&E staining along with Oil Red O staining revealed enlargement of hepatocellular volumes and increased lipid accumulation in the liver tissues of NAFLD mice compared to CD mice (Figure 1B). As expected, the liver weight and triglycerides (TG) were increased in NAFLD mice (Figure 1C,D). Notably, NAFLD mice exhibited elevated serum TG levels (Figure 1E). Furthermore, alanine aminotransferase (ALT) and aspartate aminotransferase (AST) levels were increased, which acts as an indicator of intensified liver damage in NAFLD mice (Figure 1F,G). Next, we examined multiple hepatic DNL-related genes such as sterol regulatory element binding TF 1 (*Srebf1*), *Acc1*, *Fasn*, and *Scd1* in the livers of NAFLD and CD mice using RT-qPCR. As shown in Figure 1H, expression levels of DNL-related genes increased. Then, we identified miR-192-5p expression levels in mouse liver tissues. Expression of miR-192-5p was significantly and visibly upregulated in the fatty livers of NAFLD mice versus the livers of CD mice (Figure 1I). Taken together, these results indicated that miR-192-5p expression was increased and was strongly associated with regulation of hepatic TG metabolism in mice with NAFLD.

### 3.2. Inhibition of miR-192-5p Aggravated Hepatic Steatosis in NAFLD Mice

**Figure 2:**
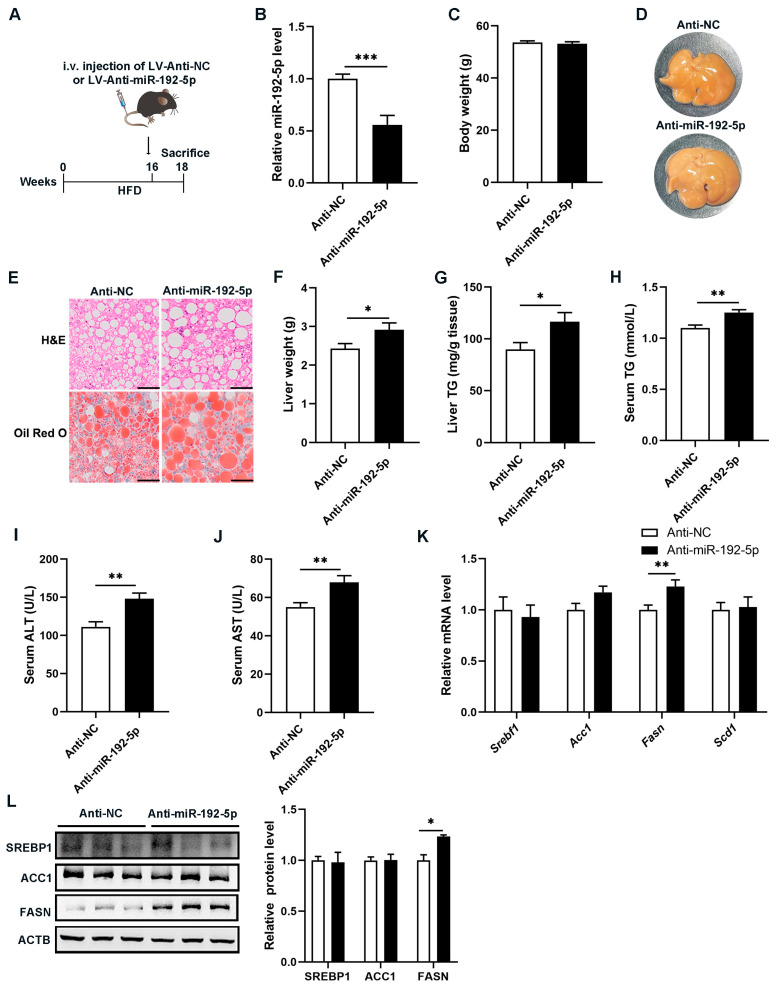
Inhibition of miR-192-5p aggravated hepatic steatosis in NAFLD mice. (**A**) Experimental scheme of NAFLD mice intravenously (i.v.) injected with lentivirus (LV)-Anti-miR-192-5p (*n* = 9) or Anti-NC (negative control) (*n* = 10). (**B**) The level of miR-192-5p in the livers of mice after two weeks of lentivirus injection. (**C**) Body weight. (**D**) Representative images of livers (*n* = 7). (**E**) Representative H&E staining (top) and Oil Red O staining (bottom) of liver tissues (Scale bar: 100 μm). (**F**) Liver weight. (**G**) Hepatic TG levels. (**H**) Serum TG levels. (**I**) Serum ALT levels. (**J**) Serum AST levels. (**K**,**L**) Relative mRNA levels (**K**) and protein levels (**L**) of DNL-related genes in livers. The data are presented as the mean ± SEM. * *p* < 0.05, ** *p* < 0.01, *** *p* < 0.001.

To further understand the biological function of miR-192-5p in hepatic lipid metabolism, we infected HFD-induced NAFLD mice with a lentivirus (LV)-miR-192-5p inhibitor or an LV-negative control (NC) via the tail vein (Figure 2A). Two weeks after injection, expression levels of miR-192-5p in mouse livers were detected using RT-qPCR. As shown in Figure 2B, the level of miR-192-5p in livers in the anti-miR-192-5p group was markedly lower than that in the anti-NC group, suggesting that the LV-miR-192-5p inhibitor had a high infection efficiency. Surprisingly, the repression of miR-192-5p in NAFLD mice resulted in aggravated liver steatosis and elevated accumulation of TG despite no significant difference in body weight (Figure 2C–E). Consistent with these findings, the liver weight and TG levels were increased in the anti-miR-192-5p mice (Figure 2F,G). Meanwhile, serum TG, ALT, and AST levels were further increased after LV-miR-192-5p inhibitor infection in NAFLD mice (Figure 2H–J). In addition, expression of *Fasn*, a key DNL enzyme, was increased in miR-192-5p inhibitor-treated NAFLD mice (Figure 2K,L). In summary, these results suggested that knockdown of miR-192-5p promoted an imbalance of TG metabolism caused by excess nutrients and aggravated hepatic steatosis in NAFLD mice.

### 3.3. Knockdown of miR-192-5p Promoted Lipid Deposition in Hepatocytes

**Figure 3:**
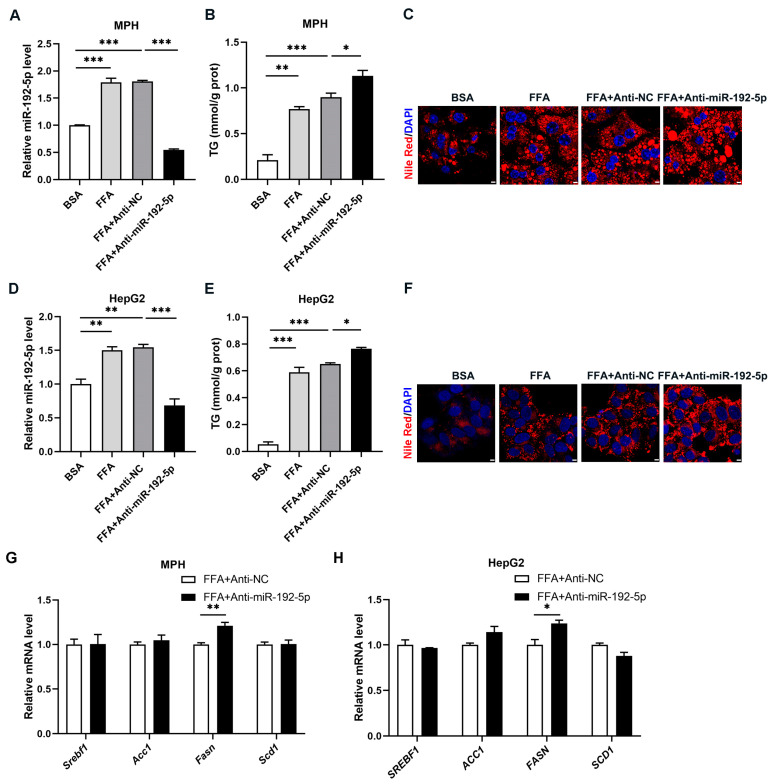
Knockdown of miR-192-5p promotes lipid deposition in hepatocytes. (**A**–**H**) Mouse primary hepatocyte (MPH) and HepG2 cells were transfected with miR-192-5p inhibitors or NC inhibitors and incubated with 0.4 mM free fatty acids (FFA, palmitic acid: oleic acid = 1:1) for 72 h (*n* = 3). The hepatocytes were treated with 0.5% bovine serum albumin (BSA) as a control (**A**–**F**). (**A**,**D**) The levels of miR-192-5p in MPH (**A**) and HepG2 cells (**D**). (**B**,**E**) TG levels in MPH (**B**) and HepG2 cells (**E**). (**C**,**F**) Lipid droplets stained with Nile Red (red) in MPH (**C**) and HepG2 cells (**F**) and DAPI (blue) for cell nuclei (Scale bar: 5 μm). (**G**,**H**) Relative expression levels of DNL-related genes in MPH (**G**) and HepG2 cells (**H**). The data are presented as the mean ± SEM. * *p* < 0.05, ** *p* < 0.01, *** *p* < 0.001.

Next, we investigated the effects of miR-192-5p on lipid metabolism to confirm the functional contribution of decreased miR-192-5p to NAFLD. Consistent with the phenotype of the NAFLD mouse model, FFA treatment for 72 h increased the expression level of miR-192-5p in both mouse primary hepatocytes and HepG2 cells (Figure 3A,D). However, miR-192-5p inhibition significantly increased TG levels in both types of cells in the presence of FFAs (Figure 3B,E). Meanwhile, Nile Red staining results showed that lipid accumulation was induced by miR-192-5p inhibitors in mouse primary hepatocytes and HepG2 cells treated with FFAs (Figure 3C,F), while mRNA levels of the *Fasn* gene were increased by miR-192-5p inhibition compared with NC in these cells (Figure 3G,H). Overall, knockdown of miR-192-5p promoted lipid deposition in hepatocytes that had undergone FFA treatment.

### 3.4. Overexpression of miR-192-5p Decreased Lipid Accumulation in Hepatocytes

**Figure 4:**
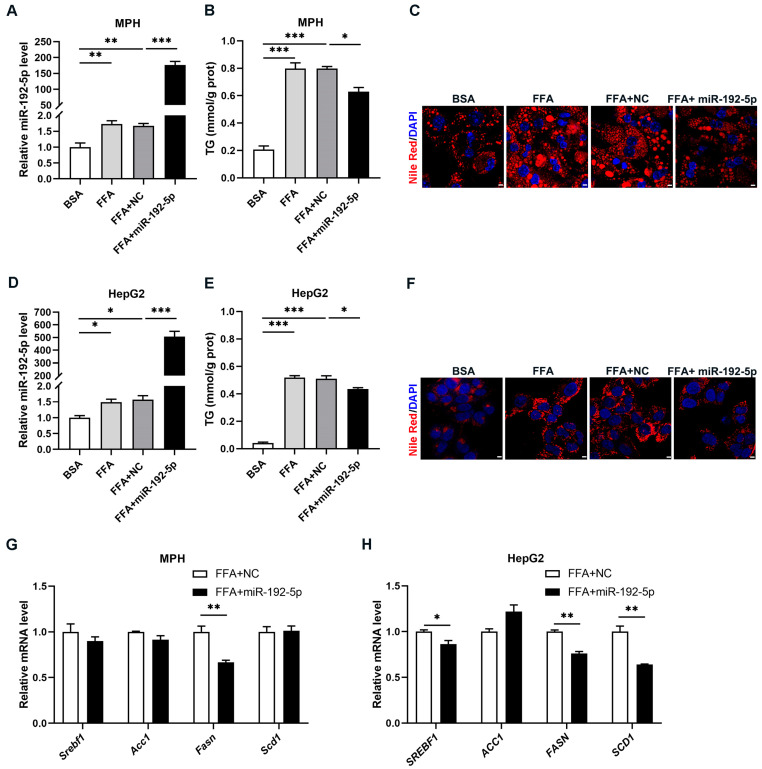
Overexpression of miR-192-5p decreased lipid accumulation in hepatocytes. (**A**–**H**) MPH and HepG2 cells were transfected with miR-192-5p mimics or NC mimics and incubated with 0.4 mM FFA for 72 h (*n* = 3). (**A**,**D**) The levels of miR-192-5p in MPH (**A**) and HepG2 cells (**D**). (**B**,**E**) TG levels in MPH (**B**) and HepG2 cells (**E**). (**C**,**F**) Lipid droplets stained with Nile Red (red) in MPH (**C**) and HepG2 cells (**F**) and DAPI (blue) for cell nuclei (Scale bar: 5 μm). (**G**,**H**) Relative expression levels of DNL-related genes in MPH (**G**) and HepG2 cells (**H**). Data are showed as mean ± SEM. * *p* < 0.05, ** *p* < 0.01, *** *p* < 0.001.

To further confirm that the function of miR-192-5p was to decrease lipid deposition in hepatocytes, we induced ectopic expression of miR-192-5p by transfecting miR-192-5p mimics into mouse primary hepatocytes and human HepG2 cells (Figure 4A,D). As shown in Figure 4B,E, miR-192-5p overexpression decreased TG levels in both cell types when treated with FFAs. Meanwhile, Nile Red staining of mouse primary hepatocytes and HepG2 cells showed that lipid deposition was remarkably lower in cells treated with miR-192-5p mimics compared to the control group. (Figure 4C,F). Furthermore, *Fasn* expression was decreased in both mouse primary hepatocytes and HepG2 cells upon overexpression of miR-192-5p. (Figure 4G,H). These data indicated that miR-192-5p overexpression decreased lipid accumulation in hepatocytes.

Taken together, these results suggest that the increased expression of miR-192-5p in the liver during the development of NAFLD is a protective mechanism of the liver against lipotoxicity. Overexpression of miR-192-5p decreased hepatic triglyceride levels, consequently reducing lipid droplet accumulation.

### 3.5. Yy1 Was a Potential Target of miR-192-5p in Hepatocytes

**Figure 5:**
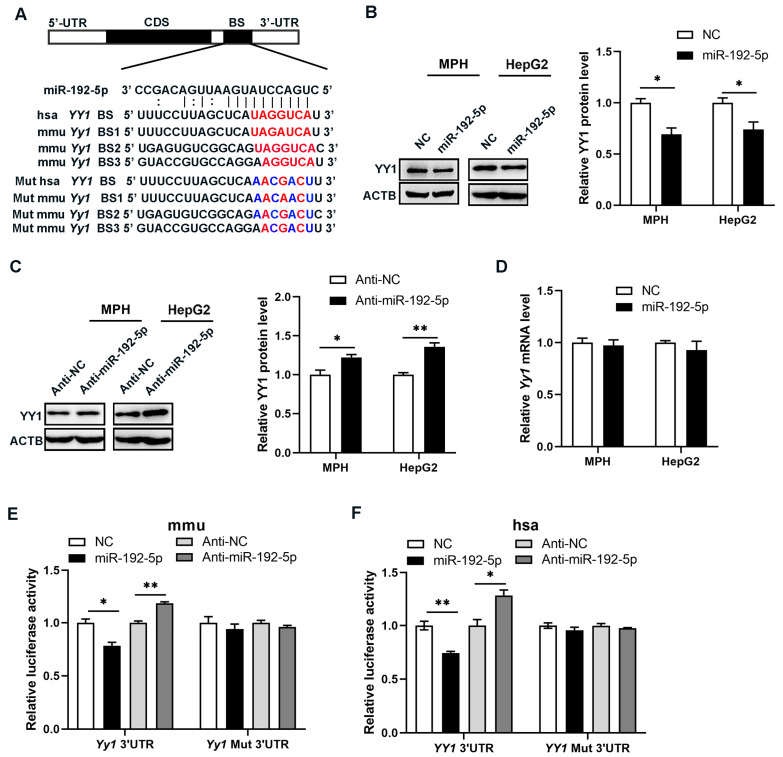
*Yy1* was a potential target of miR-192-5p in hepatocytes. (**A**) Sequences of potential binding sites (BS) of miR-192-5p within the 3′-UTR of human and mouse *Yy1*. The miR-192-5p seed-recognition sites in the *Yy1* 3′-UTR are indicated in red, and the mutant (Mut) miR-192-5p binding sites in the *Yy1* 3′-UTR are indicated in blue. (**B**,**C**) Protein levels of YY1 in MPH and HepG2 cells treated with miR-192-5p mimics (**B**) or inhibitors (**C**) revealed by Western blotting (*n* = 3). (**D**) mRNA levels of *Yy1* in MPH and HepG2 cells. (**E**,**F**) Luciferase activities of HEK293T cells transfected with a reporter vector containing the wild-type (WT) 3′-UTR or the mutant of mouse (**E**) or human (**F**) *Yy1* together with miR-192-5p mimics or inhibitors. Data are showed as mean ± SEM. * *p* < 0.05, ** *p* < 0.01.

To investigate the molecular mechanism underlying miR-192-5p’s involvement in TG metabolism, we took an in silico approach using TargetScan [34] to screen target genes of miR-192-5p. *Yy1* was identified as a potential one. As shown in Figure 5A, there was one putative binding site for miR-192-5p in the 3′-untranslated region (UTR) of human *Yy1* mRNA and three such sites in the 3′-UTR of mouse *Yy1* mRNA. To investigate the potential regulation of Yy1 by miR-192-5p, we first examined the impact of miR-192-5p on YY1 protein level in mouse primary hepatocytes and HepG2 cells. As anticipated, the protein level of YY1 was significantly reduced by the introduction of miR-192-5p mimics in both types of cells (Figure 5B and Figure S1A), whereas miR-192-5p inhibitors notably augmented the level of YY1 protein. (Figure 5C and Figure S1B). Then, to determine the level at which miR-192-5p regulated *Yy1* expression, we also examined the expression of *Yy1* mRNA. Intriguingly, our finding revealed that overexpression of miR-192-5p did not affect mRNA levels of *Yy1* in mouse primary hepatocytes or HepG2 cells (Figure 5D). These data suggested that miR-192-5p specifically regulated YY1 protein expression at the post-transcriptional level. To ascertain whether miR-192-5p directly regulated *Yy1* expression by binding with the *Yy1* 3′-UTR, the 3′-UTR of human or mouse *Yy1* containing presumed miR-192-5p binding sites was fused downstream of the firefly luciferase gene in reporter plasmids, which were then independently transfected into 293T cells along with a transfection control plasmid (β-gal) and miR-192-5p mimics or inhibitors. As anticipated, overexpression of miR-192-5p resulted in a reduction of luciferase reporter activity by approximately 20%, whereas inhibition thereof increased reporter activity 1.2-fold compared with cells transfected with control inhibitors (Figure 5E,F). Furthermore, we mutated the corresponding complementary sites in the 3′-UTR of *Yy1* to disrupt miR-192-5p binding. The mutated luciferase reporter was unaffected by miR-192-5p mimics or inhibitors. In conclusion, these results demonstrated that miR-192-5p inhibited *Yy1* expression by binding to the 3′-UTR of *Yy1*.

### 3.6. MiR-192-5p Inhibited Hepatic Triglyceride Synthesis in NAFLD Mice by Regulating the YY1/FASN Pathway

**Figure 6:**
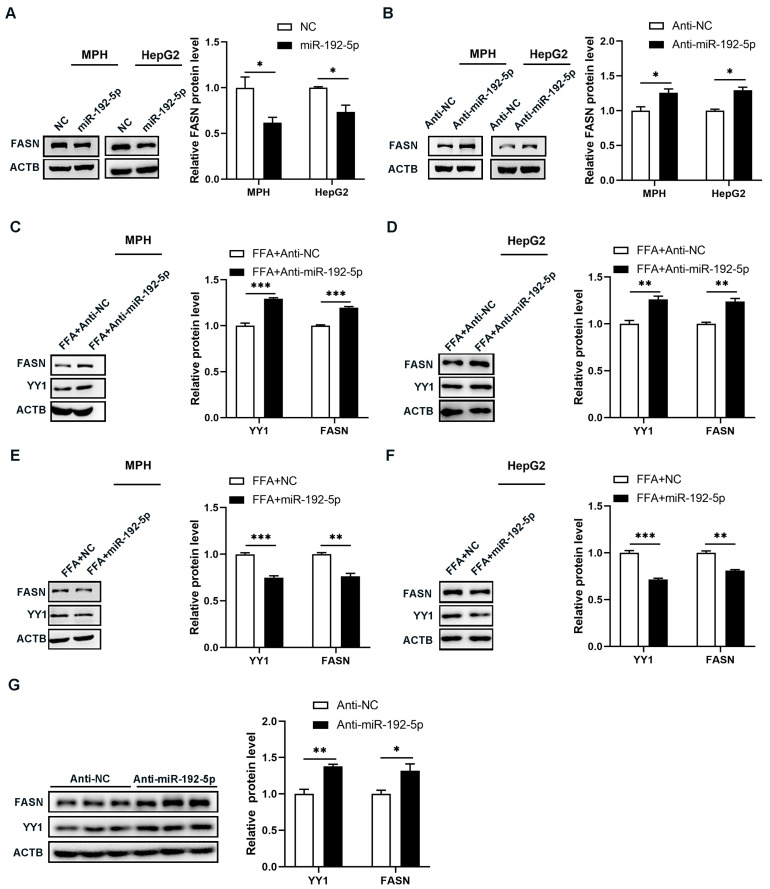
MiR-192-5p inhibited hepatic triglyceride synthesis in NAFLD mice by regulating the YY1/FASN pathway. (**A**,**B**) Protein levels of FASN in MPH and HepG2 cells transfected with miR-192-5p mimics (**A**) or inhibitors (**B**) for 48 h. NC mimics or inhibitors as a control (*n* = 3). (**C**,**D**) Protein levels of YY1 and FASN in MPH (**C**) and HepG2 cells (**D**) treated with miR-192-5p inhibitors and incubated with 0.4 mM FFA for 72 h. (**E**,**F**) Protein levels of YY1 and FASN in MPH (**E**) and HepG2 cells (**F**) treated with miR-192-5p mimics and incubated with 0.4 mM FFA for 72 h. (**G**) Protein levels of YY1 and FASN in the livers of NAFLD mice injected with LV-Anti-miR-192-5p or LV-Anti-NC for two weeks. Data are presented as mean ± SEM. * *p* < 0.05, ** *p* < 0.01, *** *p* < 0.001.

It has been reported that YY1 promotes hepatic lipogenesis by activating the expression of FASN [28]. In order to investigate whether miR-192-5p decreased lipid accumulation in hepatocytes by regulating the YY1/FASN pathway, we further examined protein levels of FASN in both mouse primary hepatocytes and HepG2 cells after expressing miR-192-5p ectopically. The results show that miR-192-5p downregulated FASN protein levels (Figure 6A and Figure S1A), while miR-192-5p inhibition resulted in increased FASN expression in both cell types (Figure 6B and Figure S1B). In vitro, inhibition of miR-192-5p led to increased YY1/FASN protein expression levels in hepatocytes treated with FFAs (Figure 6C,D and Figure S1C), while overexpression of miR-192-5p significantly reduced their protein expression (Figure 6E,F and Figure S1D). Furthermore, YY1 and FASN protein levels in the livers of NAFLD mice were further increased after LV-miR-192-5p inhibitor infection (Figure 6G). In summary, these results indicated that miR-192-5p inhibited hepatic TG synthesis in NAFLD mice by regulating the YY1/FASN pathway.

## 4. Discussion

NAFLD is commonly linked to dysfunction in lipid metabolism [1]. However, the precise molecular mechanisms responsible for this association have not been fully understood. Recent studies have found that miRNAs are important regulators involved in the development of NAFLD [35,36,37]. In this study, we demonstrated that miR-192-5p inhibited hepatic fatty acid synthesis by targeting *Yy1*, thereby reducing TG accumulation in NAFLD. Our findings will provide new insight into therapy for NAFLD.

MiR-192-5p is abundant in the liver [19]. Our study found that miR-192-5p expression levels were significantly elevated in NAFLD mice and FFA-treated hepatocytes. This finding is in agreement with previous studies that have shown a significant increase in miR-192-5p levels in both the serum and livers of individuals with NAFLD compared to healthy individuals [23,38]. However, the exact impact and mechanisms of miR-192-5p in the development of NAFLD are not well understood. To investigate its role in NAFLD, we suppressed miR-192-5p expression in the livers of NAFLD mice by administering an LV that overexpressed anti-miR-192-5p. Interestingly, inhibition of miR-192-5p resulted in increased accumulation of hepatic TGs and aggravated hepatic steatosis in NAFLD mice. In vitro, inhibiting miR-192-5p also increased lipid droplet accumulation in hepatocytes. Moreover, overexpressing miR-192-5p reduced TG levels. Taken together, our findings indicated that hepatic miR-192-5p, a protective miRNA, was adaptively elevated and thereby reduced hepatic lipid accumulation during NAFLD development.

An increase in DNL, the process by which the hepatic non-lipid metabolite acetyl-CoA is synthesized into FAs, is a major pathogenic mechanism of NAFLD [39,40]. In this study, the expression levels of genes involved in DNL, including *Srebf1*, *Acc1*, *Fasn*, and *Scd1*, were evaluated. We observed significantly increased FASN expression levels in NAFLD mice and FFA-treated hepatocytes after inhibition of miR-192-5p. Moreover, miR-192-5p overexpression led to a reduction in FASN levels in hepatocytes. FASN has been recognized as an appealing therapeutic target for NAFLD [41]. Several effective FASN inhibitors have been reported for potential utility in metabolic disease [42,43,44,45]. Our findings suggested that miR-192-5p regulates FA synthesis via FASN. We attempted to find miR-192-5p binding sites within the *Fasn* gene using TargetScan; however, none were identified. This suggested that miR-192-5p regulated FASN expression in indirect ways, possibly involving TFs.

YY1 is a widely distributed TF in mammalian cells, with dual roles as a transcriptional activator and repressor [24,46]. It has been reported that YY1 expression is markedly increased in patients with NAFLD and directly promotes hepatic steatosis via activation of hepatic FA synthesis [29]. In our NAFLD mouse model, the YY1 expression level in the liver tissues of mice fed with HFD was significantly higher than that of mice fed with CD (Figure S2). Transcriptome sequencing analysis of *Yy1* knockout HepG2 cells revealed that downregulated genes are associated with various lipid metabolism processes, including FA metabolism, lipid synthesis, lipid transport, steroid synthesis, and cholesterol metabolism [47]. Moreover, YY1 has been found to directly promote the expression of FASN by binding to its promoter region [28]. In this study, we used the miRNA target prediction software to screen *Yy1* as a target gene of miR-192-5p. Moreover, miR-192-5p has been reported to inhibit the growth of bladder cancer cells by targeting YY1 [48]. However, it remains unclear whether miR-192-5p/YY1 is involved in regulating hepatic lipid metabolism. By inhibiting miR-192-5p, we observed an increase in the expression of YY1 and FASN in NAFLD mice. In vitro, miR-192-5p decreased the expression of YY1/FASN and improved the accumulation of lipid droplets in hepatocytes.

## 5. Conclusions

In the present study, we provided new evidence that miR-192-5p was involved in the pathogenesis of NAFLD and that it regulated hepatic lipogenesis by targeting the YY1/FASN pathway. Our findings emphasized that promoting miR-192-5p expression favored the maintenance of hepatic lipid metabolic homeostasis, thus providing a promising therapeutic modality for the treatment of NAFLD.

## Data Availability

The data presented in this study are available on request from the corresponding author.

## Supplementary Materials

The following supporting information can be downloaded at: https://www.mdpi.com/article/10.3390/biom14010034/s1, Figure S1: *Yy1* was a potential target of miR-192-5p and miR-192-5p inhibited hepatic triglyceride synthesis by regulating the YY1/FASN pathway; Figure S2: YY1 expression was upregulated in the liver of NAFLD mice; Table S1: Primers for qRT-PCR.
