# Supplementary material for: MiR-192-5p Ameliorates Hepatic Lipid Metabolism in Non-Alcoholic Fatty Liver Disease by Targeting Yy1
Source: Biomolecules. 2023 Dec 26;14(1):34. doi: 10.3390/biom14010034 (PMC10813355; doi:10.3390/biom14010034)
Supplement: Supplementary file 1 [file biomolecules-14-00034-s001.zip › biomolecules-2722436-supplementary.pdf]

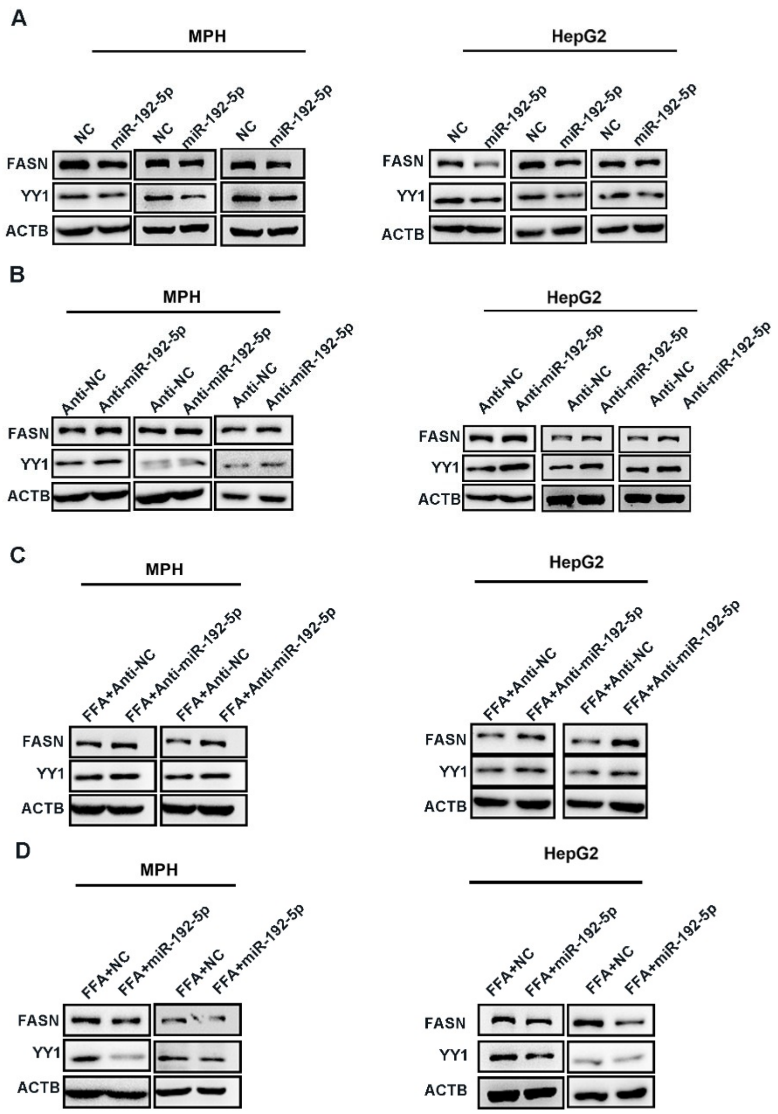

Figure S1. *Yy1* was a potential target of miR-192-5p and miR-192-5p inhibited hepatic triglyceride synthesis by regulating the YY1/FASN pathway. (A and B) Protein levels of YY1 and FASN in MPH and HepG2 cells treated with miR-192-5p mimics (A) or inhibitors (B) revealed by Western blotting. (C and D) Protein levels of YY1 and FASN in MPH and HepG2 cells treated with miR-192-5p inhibitors (C) or miR-192-5p mimics (D) and incubated with 0.4 mM FFA for 72 h.

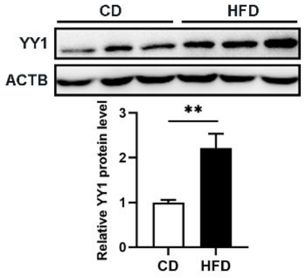

Figure S2. YY1 expression was upregulated in the liver of NAFLD mice, ** $p < 0.01$.

Table S1. Primers for qRT-PCR

Gene	Forward primer (5′-3′)	Reverse primer (5′-3′)
Mouse β-Actin	TATCGCTGCGCTGGTCGTC	CCACGATGGAGGGGAATACAG
Mouse Srebf1	GGAGCCATGGATTGCACATT	GGCCCGGGAAGTCACTGT
Mouse Acc1	GGAGATGTACGCTGACCGAG	TACCCGACGCATGGTTTTCA
Mouse Fasn	GGCCCCTCTGTTAATTGGCT	GGATCTCAGGGTTGGGGTTG
Mouse Scd1	CCTCCGGAAATGAACGAGAGA	CTGATAGGTGGGGTCGTGAA
Mouse Yy1	TGCCCTCATAAAGGCTGCAC	CTCTCAACGAACGCTTTGCC
Human β-ACTIN	GGGAAATCGTGCGTGACATT	GGAACCGCTCATTGCCAAT
Human SREBF1	ACAGTGACTTCCCTGGCCTAT	GCATGGACGGGTACATCTTCAA
Human ACC1	CACCAGTTTTGCATTGAGAAC	TACGCTGTTGAGTTCATAGGC
Human FASN	TATGAAGCCATCGTGGACGG	GAAGAAGGAGAGCCGGTTGG
Human YY1	GACACCCTCTACATCGCCAC	TGTGGTCTCGATGGTCTCCA
