# Supplementary figures and images for: MiR-192-5p Ameliorates Hepatic Lipid Metabolism in Non-Alcoholic Fatty Liver Disease by Targeting Yy1
Source: Biomolecules. 2023 Dec 26;14(1):34. doi: 10.3390/biom14010034 (PMC10813355; doi:10.3390/biom14010034)

mouse Primary hepatocyte (MPH)

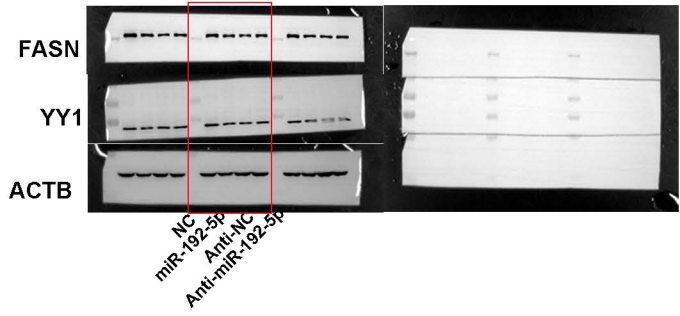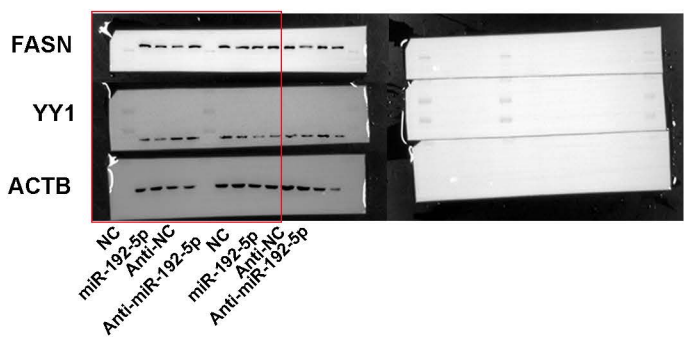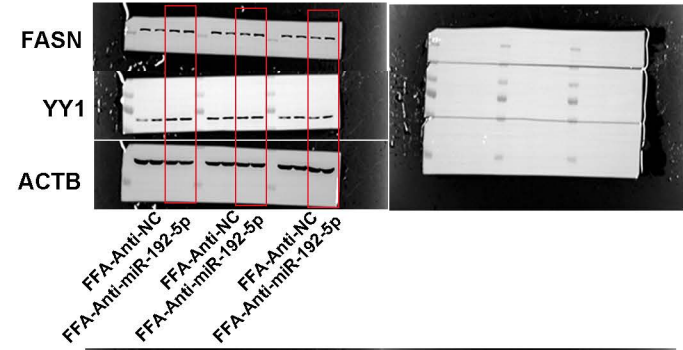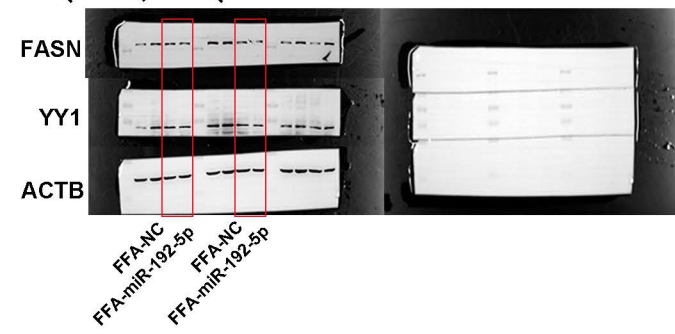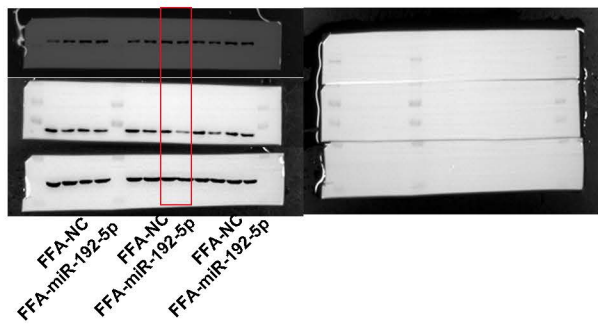

HepG2

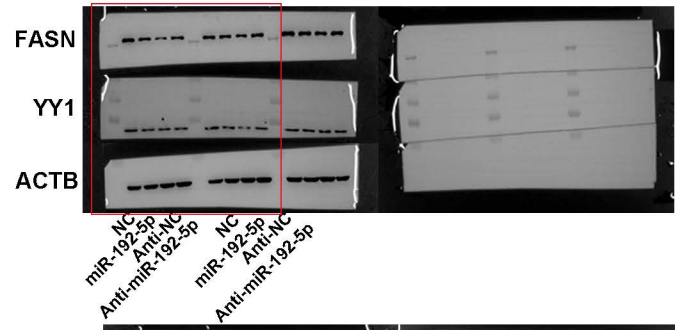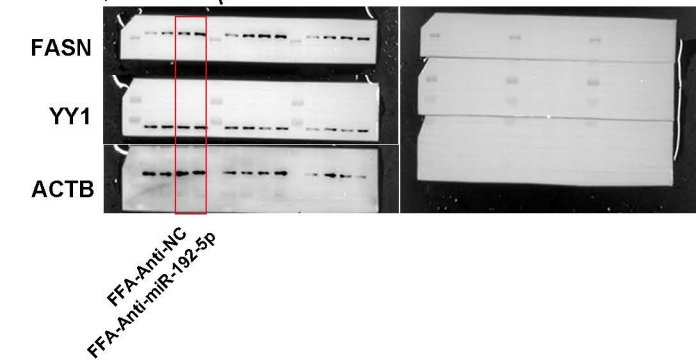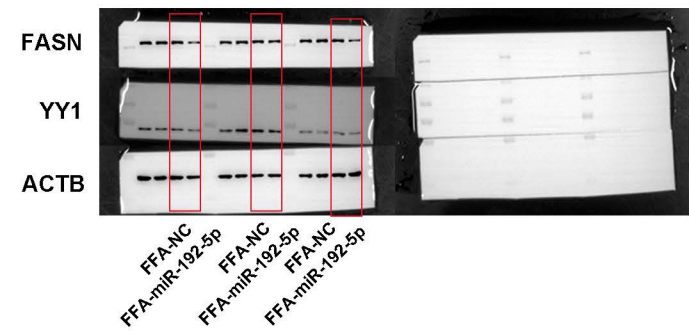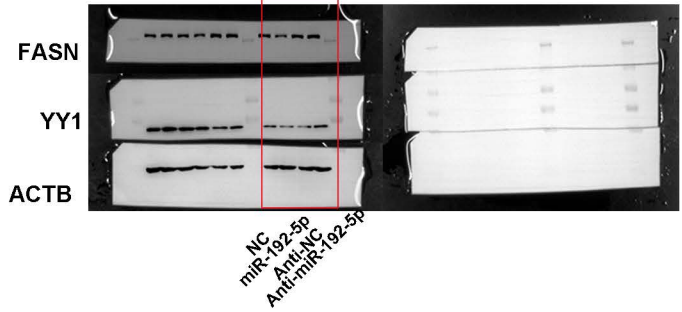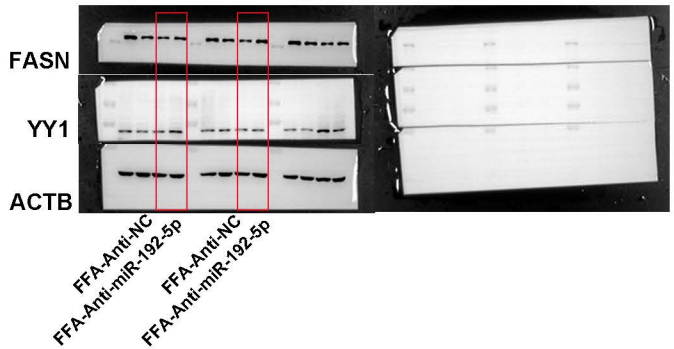

Mice

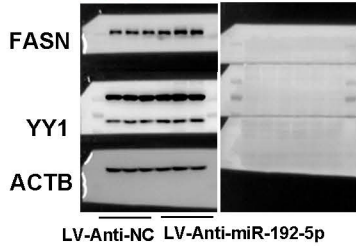

Supplement: Supplementary file 1 [file biomolecules-14-00034-s001.zip › biomolecules-2722436-original-images.pdf]
